# Strength and Conditioning Programs to Increase Bat Swing Velocity for Collegiate Baseball Players

**DOI:** 10.3390/sports11100202

**Published:** 2023-10-16

**Authors:** Ryosuke Haruna, Tatsuo Doi, Daiki Habu, Shinya Yasumoto, Nobuko Hongu

**Affiliations:** 1 Department of Nutrition, Graduate School of Human Life and Ecology, Osaka Metropolitan University, 3-3-138, Sugimoto, Sumiyoshi-ku, Osaka 558-8585, Japan; haruna@dsmi.jp (R.H.); habu@omu.ac.jp (D.H.); 2 Dynamic Sports Medicine Institute, 1-10-28, Nishi-Shinsaibahi, Chuo-ku, Osaka 542-0086, Japan; loco1952@dream.jp (T.D.); yasumoto@dsmi.jp (S.Y.)

**Keywords:** baseball, bat swing velocity, strength and conditioning, training, batting performance

## Abstract

Bat swing velocity (BSV) is an imperative element of a successful baseball hitting performance. This study aimed to investigate the anthropometric and physiological variables associated with BSV and explore strength and conditioning programs to increase BSV in collegiate baseball players. Seventy-eight collegiate baseball players (mean age ± SD, 19.4 ± 1.0 years) participated in this study. Maximum BSV (km/h) was measured using Blast Baseball (Blast Motion Inc., Carlsbad, CA, USA). The anthropometric and physiological variables measured were height, body mass, lean body mass, grip strength, back muscle strength, the 30 m sprint, standing long jump, and backward overhead medicine ball throwing. Analysis using Pearson’s product-moment correlation coefficient showed a weak but significant positive correlation between all anthropometric measurements to BSV. Significant relationships existed between physiological variables of hand grip, back muscle strength, and backward overhead medicine ball throwing, but not the standing long jump and 30 m sprint. These data show that BSV is related to anthropometric and physiological variables, particularly upper and lower body strength and full-body explosive power. Based on the results of this study, we designed examples of sound training programs to increase BSV. Strength and conditioning coaches may want to consider using this information when designing a training program for collegiate baseball players.

## 1. Introduction

Baseball is a popular sport around the world. Recently, many Japanese professional baseball players have played in the U.S. Major League Baseball (MLB), which is the highest level of professional baseball [1]. Their great performance draws the attention, excitement, and interest of basketball fans watching or playing baseball. This sport is played between two teams (i.e., batting and fielding teams). The objective of the game is to hit a pitched ball out of reach of the fielding team and to score a `run’. The team that scores the most runs wins the game. To hit a ball a long distance, hitters need to have a fast bat swing velocity (BSV). Three benefits of increased BSV are (1) increased decision time (i.e., the time between recognizing the pitched ball and deciding to swing the bat), (2) decreased swing time, and (3) increased batted ball velocity [2]. The BSV is correlated with successful hitting; thus, it has been regarded as a major indicator of batting performance [2,3,4,5,6,7]. The BSV is produced by the hitter’s rotation motion around the longitudinal axis [8].

Previous research has shown that torso rotation strength helps increase BSV [9]. This baseball-specific rotational motion is achieved through the sequential recruitment of large muscles from the legs up to the hips and torso, which transfer momentum to the upper body segments [2,9,10]. Katsumata [11] explained the structure of the hitting movement as producing a “ground reaction force”, a powerful bat swing, and timing. A baseball batter takes a step with his front foot in the direction of a pitcher and shifts weight forward to the front foot, fixing the front foot on the ground for hip and upper body rotation. Researchers have reported significant correlations between lower body strength [9,11,12], torso rotational strength [13], and BSV in youth and collegiate baseball players. Kohmura et al. [14] showed that the strength of the lower extremity and back muscle is an important component of BSV in Japanese collegiate baseball players.

Strong associations have been identified that increasing muscle strength results in overall muscle volume in various resistance training programs [15,16]. Thus, increasing BSV may be positively correlated not only with muscle strength and power but also with increasing muscle volume [17]. Hoffman et al. [18] assessed anthropometric (i.e., height, body mass, % body fat, and lean body mass) and physiological (i.e., grip strength, vertical jump power, 10-yard sprint speed, and agility) variables at the beginning of spring training during a 2-year period in 343 professional baseball players. The results of this study indicated that lean body mass, grip strength, speed, and lower-body power were correlated with baseball-specific performance variables (i.e., home runs, total bases, slugging percentage, and stolen bases). Agility, speed, and lower-body power provided the greatest predictive power to baseball-specific performance variables. Szymanski et al. [7] also investigated anthropometric, physiological performance variables (i.e., rotational power, rotational strength, vertical jump, estimated peak power, upper and lower body strength, and angular hip and shoulder velocities) and the BSV of high-school baseball players before and after performing 12 weeks of resistance training during the off-season (fall semester). They reported that anthropometric variables were related to BSV, suggesting that a taller, heavier, and greater lean body mass in high-school baseball players had a faster BSV. Based on the results of this and their previous studies, the practical application for generating hitting power was recommended. Comprehensive strength and conditioning programs were designed, such as full body resistance training and rotational medicine ball exercises, which were incorporated into regular practices of various baseball fielding tasks, like hitting and throwing [19]. To improve hitting performance, baseball players commonly performed a hitting drill [20,21]. DeRenne et al. [22] suggested the combination of weight training and hitting drill protocols on BSV that could be included, such as (1) batting practice in their on-field preseason and (2) dry swing protocol in the weight training sessions of collegiate baseball players.

Baseball coaches and players use baseball-specific training to become better baseball players. Baseball-specific training is training that helps baseball players to apply their strength and power to the batting motion. The main purpose of this training is two-fold: the first is the training of “on and off” muscle contractions and learning which parts of the muscles contract and relieve at any given time in the batting motion. The second purpose is training to create momentary power when needed. This training enhances the appropriate muscle contraction and strength at the “right time”, creating explosive power that transfers to BSV. The batting motion begins with the lower limbs and transfers this power in a series of steps to the torso, upper limbs, and then to the bat [9,10]. Now, baseball players vary widely in terms of individuality. We hypothesize that there are a few types of baseball players in terms of body composition, strength, and power for building individualized training content. Therefore, the purpose of the present study was to investigate the relationship between anthropometric, physiological variables and BSV in collegiate baseball players. Additionally, the novelty of the present study was to present some examples of baseball-specific strength and conditioning training based on our findings on types of baseball players. These baseball-specific training strategies could be implemented to optimize competitive performance in collegiate baseball players.

## 2. Materials and Methods

### 2.1. Study Design

A correlational study design was used to examine a possible relationship between various anthropometrics (i.e., height, body mass, % body fat, lean body mass) and physiological performance (i.e., muscle strength, total and lower body power, and agility) to BSV. We hypothesized that anthropometric and physiological performance variables could be an effective predictor of greater BSV. All testing was conducted by certified strength and conditioning specialists during the off-season training period (December 2022). All procedures were approved by the Osaka Metropolitan University Institutional Ethical Review Board (IRB # 22-65).

### 2.2. Participants

Seventy-eight male baseball players were recruited through verbal and email contact. Their team was a member of the Hanshin University Baseball League, which is a Division I collegiate baseball league in Japan. All participants met the following inclusion criteria: male collegiate baseball players aged 18 to 25 years free of any musculoskeletal injuries and medical conditions within the preceding 6 months that prevented participation in baseball. Participants were excluded if they had a current injury preventing them from competing in baseball. Each participant received the purpose of the study and measurement details, risks, and benefits prior to participating in the experiments. Written informed consent was obtained from all participants. Participants completed a short questionnaire, which included their age, the year of experience that they played baseball, and positions of play.

### 2.3. Anthropometric and Body Composition Assessment

Body weight (kg) and height (cm) were recorded to the nearest decimal to calculate body mass index (BMI) (kg/m^2^). Body weight, body fat, and lean body mass were measured with a single-frequency bioelectrical impedance analyzer (BC-118, Tanita, Tokyo, Japan). During the assessment, the participants wore light baseball training wear without socks. Participants stood on the measurement stand on a platform with normal breathing and no movement during the measurement. Height was measured with a stadiometer to the nearest 0.5 cm.

### 2.4. Batting Performance Test

**Figure 1:**
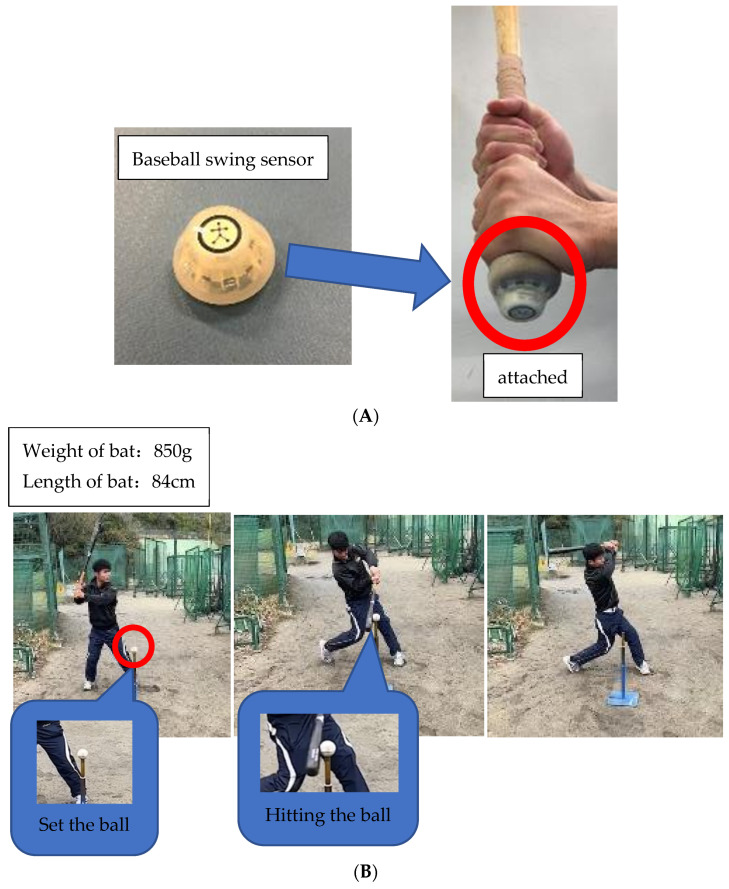
(**A**) Baseball swing sensor attached to the tail of a bat. (**B**) Measurements of BSV using a Blast Motion baseball swing sensor.

Before testing, each participant performed warm-up swings and dynamic stretches of the arms, lower back, legs, and knees for about 3 min. Dynamic stretches increase muscle temperature, expand the range of motion, and enhance muscle strength [23]. Swing velocity was measured using a Blast Motion baseball swing sensor (Blast Motion Inc., Carlsbad, CA, USA), which was mounted on the tail of a bat (Figure 1A). Each participant’s swing speed data were calculated from the displacement of the coordinates of a position away from the bat head in the direction of the tail [24]. The accuracies of swing speed measured using the Blast Motion sensor have shown high reliability with an optical motion capture system, suggesting its usefulness for evaluating differences in swing speeds among collegiate players [25]. Each participant stood in a simulated batter’s box, facing a ball and rotating their torso while swinging. A baseball was attached to a tee stand on a home plate to simulate a real hitting situation. Participants were instructed to hit the ball straight, keeping the bat parallel to the ground, swing as hard as they could, and ensure swinging with follow through, i.e., letting the bat slow down as it crossed in front of their chest (Figure 1B). If they had an air swing or missed the timing, participants were asked to reattempt a hitting trial. After three maximal effort attempts, the highest score in kg/h was recorded.

### 2.5. Physical Performance Tests

#### 2.5.1. Handgrip Testing

Maximum grip strength was assessed using a GRIP-D (Takei Scientific Instruments Co., Ltd., Niigata, Japan) [26]. The participant’s right and left hands were assessed. The width of the dynamometer handle was adjusted according to the individual’s preference. Each participant stood still and upright, looking ahead, and the hand to be tested could not be touched with the thigh or any part of the body. Participants were instructed to perform a maximum effort attempt. During testing, verbal encouragement was given to the participants to obtain their best score. Four measurements were taken, and each one was supposed to last a few seconds [27,28]. Participants performed the first 2 measurements in a row: one with the dominant hand and the other with the non-dominant hand. Then, one minute of rest was allotted before the 2 measurements were repeated. The highest scores of both hands were recorded in kilograms (kg).

#### 2.5.2. Back Muscle Strength Testing

**Figure 2:**
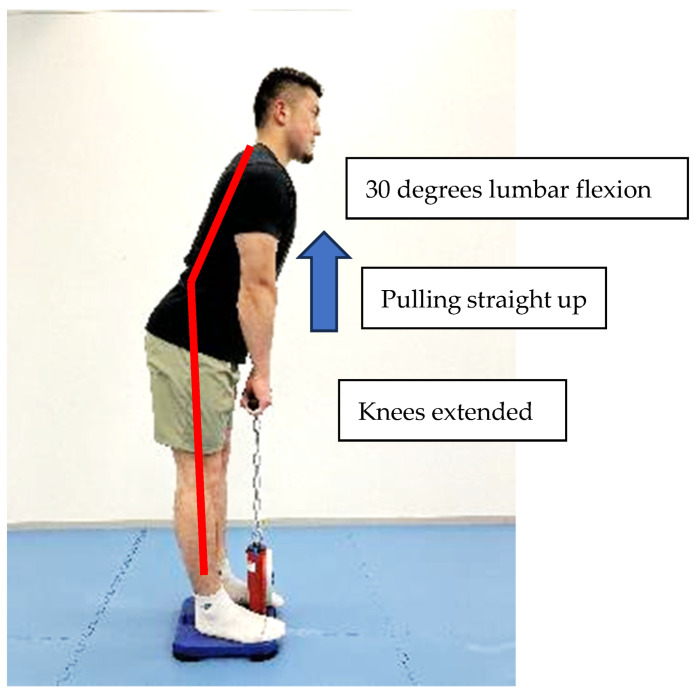
Back muscle strength measurement.

We determined back muscle strength from the maximal isometric strength of the trunk muscles in a standing position with 30 degrees lumbar flexion [29] and the knees extended using a digital back muscle strength meter, BACK-D (Takei Scientific Instruments Co., Ltd., Niigata, Japan) [30] (Figure 2). We confirmed the intra-rater reliability of back muscle maximum strength by measuring 11 healthy participants three times. Intra-rater reliability was high with ICC of 0.980. Each study participant performed one maximal attempt, and data were recorded in kg. In case a participant wanted to repeat the test, he was allowed to repeat the test after a minute’s break. Only one participant tried to repeat the test, but data were not recorded in this study.

#### 2.5.3. Standing Long Jump

The standing long jump is a simple, reliable test to measure lower leg power [31,32]. Participants stood with both feet behind the starting line. Subsequently, they were encouraged to jump as far as possible, landing with both feet. They were not allowed to use arm swings during the test. The maximal distance from the starting line to the landing point at heel contact was measured in centimeters. Participants performed two attempts [33,34]. The longest distance was used for data analysis.

#### 2.5.4. The 30 m Sprint

Speed was assessed through a 30 m sprint test [35]. Sprint times were measured using a 30 m straight track with markers for the starting and finishing lines. Dash measurements were performed using a photoelectric tube sensor, Dashr Blue (Dashr-Motion Performance Systems Inc., Lincoln, NE, USA). Participants performed warm-ups that included 2~3 min of light jogging and various stretching exercises, including the lower legs, thighs, lower back, and arms. All participants ran one trial. In case a participant had trouble at the start or finished the test, the participant was allowed a re-test trial. The starting position was a sideways standing start with the right side of the body toward the direction of running.

#### 2.5.5. Backward Overhead Medicine Ball Throwing

A 3 kg medicine ball (LIND SPORTS Inc, Osaka, Japan) was used to determine the full-body throwing power combining the upper and lower body [36,37,38,39]. At the beginning of the test, participants stood with their feet shoulder-width apart and held the medicine ball with arms straight in front of the body. Then, the hips and knees were flexed to perform the countermovement before extending with rapid hyperflexion of the shoulders to throw the ball overhead as far as possible. The distance between the participant and the point where the ball first landed on the ground was measured in meters. Each throw was recorded to the nearest 10 cm. Since our study participants were regularly engaged in medicine ball throwing as part of their off-season strength and conditioning program, participants performed only two throws instead of 5 or 6 practice throws, as recommended by Duncan et al. [39]. The longest distance was used for data analysis.

### 2.6. Statistical Analysis

Data are presented as group mean values ± SD. After confirming normality with the Shapiro–Wilk test, Pearson’s product-moment correlation coefficient was used to evaluate the correlation between BSV and each measurement item. Items that did not show normality were evaluated using Spearman’s rank correlation coefficient. Multiple regression analysis on BSV was performed using linearized equations for all subjects. In addition, we examined whether there were differences in all the measured variables (i.e., anthropometric and physical performance) for BSV. BSV was divided into three groups: Fast BSV (112.0 ± 4.0 km/h), Middle BSV (104.9 ± 1.4 km/h) and Slow BSV (98.8 ± 2.8 km/h). Comparisons of the groups were performed using the Kruskal–Wallis test and the Dunn-Bonferroni correction was used for the post hoc test. A *p*-value less than 0.05 was considered statistically significant. All statistical analyses were performed using SPSS (IBM SPSS statistics, Version 28, Armonk, NY, USA).

### 2.7. Ethics

This study was conducted in accordance with the tenets of the Declaration of Helsinki. Ethical approval was obtained from the Ethics Committee of the School of Human Life Science, Osaka City University (approval number 21-42, approved on 13 October 2021).

## 3. Results

A total of 78 male collegiate baseball players were included in the study. The results of anthropometric, batting performance, and physical performance tests are presented in Table 1. The mean age of participants was 19.4 ± 1.0 years (mean ± SD). All were identified as Asian, living in Japan, and they had been playing baseball since childhood. The mean BSV (km/h) of all players (n = 78) was 105.2 ± 6.1. The range of BSV was 90.5 km/h to 123.3 km/h.

**Figure 3:**
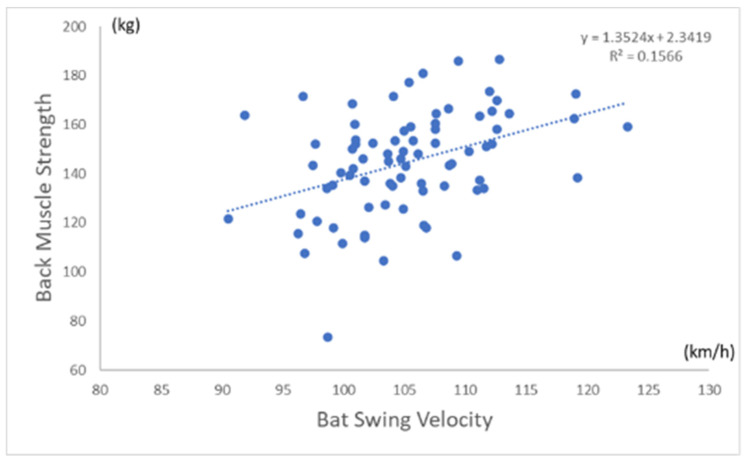
Correlation between BSV and back muscle strength. Scatterplots to visualize relationship between BSV and back muscle strength (Figure 3) and lean body mass (Figure 4). Blue dot indicates each baseball player.

**Figure 4:**
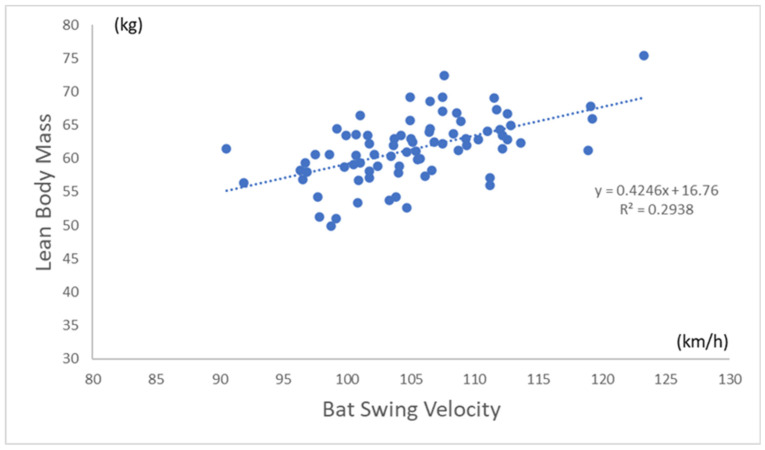
Correlation between BSV and total lean body mass. Scatterplots to visualize relationship between BSV and lean body mass. Blue dot indicates each baseball player.

Table 2 shows the correlation between BSV and each measurement. The BSV had a significant positive correlation with all anthropometric measurements (height, *p* = 0.002 and all others, *p* < 0.001). For physical performance test variables, the BSV was significantly correlated with only grip strength (*p* = 0.003), back muscle strength (*p* < 0.001), and backward overhead medicine ball throwing (*p* = 0.012). Figure 3 shows the correlation between BSV (km/h) and back muscle strength (kg). Figure 4 shows the correlation between BSV (km/h) and the total lean body mass (kg) in all participants. Table 3 shows multiple regression analyses for factors influencing BSV. The BSV had a significant positive correlation with only lean body mass (*p* < 0.001) and back muscle strength (*p* = 0.032).

Anthropometric and physical performance comparisons for the three levels (i.e., Fast, Middle, and Slow) of BSV are shown in Table 4. The Fast BSV group was significantly heavier in body weight and lean body mass than the Middle and Slow BSV groups. The Fast BSV group was significantly taller, had greater upper limb lean body mass (i.e., dominant hand) and lower limb lean body mass (i.e., stride leg) than the Slow BSV group. No differences were observed in anthropometric variables between the Middle and Slow BSV groups.

The Fast BSV group had significantly greater hand grip and back muscle strength than the Slow BSV group. The Middle BSV group had a greater hand grip than the Slow BSV. No significant differences were noted in the standing long jump or 30 m sprint in any BSV group.

## 4. Discussion

Baseball players with greater strength, power, and lean body mass had a faster BSV. A faster BSV is crucial for hitting a baseball, enabling it to travel at a greater distance [2,7,31]. Batters attempted to achieve a faster BSV by producing force from the ground up through their legs, hips, torso, and upper body. They rotated their trunk rapidly with full effort. However, although this batting action is based on physiological factors and kinetic links to successful batting performance, we speculate that there is still a need for baseball-specific strength and conditioning workout programs for the better performance of baseball players. The objective of our study was to report significant relationships between anthropometric and physiological variables and BSV. In addition, based on our findings, we propose some baseball-specific strength and conditioning workout programs for increasing BSV that could link to successful performance among collegiate baseball players.

**Figure 5:**
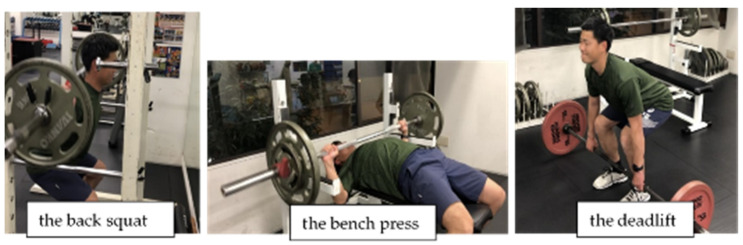
The big three (the back squat, the bench press and the deadlift).

In our study, Table 2 shows the significant relationships between anthropometry, physiological performance variables, and BSV. These results align with previous research findings of contributing factors for increasing BSV. However, the correlation coefficients were relatively weak. In Table 2, the largest *r* value was 0.594, meaning that only approximately 35% of variations between the variables can be explained in our participants. Table 3 shows that BSV had a significant positive correlation with only lean body mass and back muscle strength. When we looked at the three levels (i.e., Slow, Middle, Fast) of BSV groups and anthropometric measurements, the Fast BSV (112.0 ± 4.0 km/h) group was significantly heavier in body weight and lean body mass and taller in height than the Middle (104.9 ± 1.4 km/h) and Slow BSV (98.8 ± 2.8 km/h) groups. These findings are similar to previous studies that report significant relationships between anthropometry, physical performance, and BSV, suggesting a relationship between lean body mass, strength, and power to BSV. The findings of this current study are in agreement with other studies reporting collegiate baseball players with greater lower body strength, rotational strength, and rotational power corresponding to faster BSV [8,9,11,12,13,14]. Szymanski et al. mentioned how, resistance training, such as the big three (i.e., the back squat, the bench press, and the deadlift, see Figure 5) play “a part of in the complete development of a baseball player, as it relates to batting velocity” based on their and other correlation studies between anthropometric and physiological variables and BSV [7]. The results for the three levels of BSV groups also indicate that there may need to be more individualized strength and resistance training to improve their BSV.

Based on the findings of both previous and our studies, we propose the following training recommendations for these three BSV groups.

*Slow and Middle BSV groups:*

Slow and Middle BSV groups had weaker handgrip strength (Table 4). The Slow BSV group had lower back muscle strength than Fast BSV, 12.3 ± 1.0 m vs. 13.7 ± 1.7 m, respectively. Our recommended strategy is strengthening the forearm muscles using bat swing training. Strengthening the forearm muscles could improve handgrip strength [40]. However, to improve BSV, strengthening the forearm muscles alone may not be sufficient. The Fast BSV players have their bats controlled by the radial/ulnar deviation action of the wrist joint on the top-hand side as the ball impacts the bat. Thereby, they can transfer a large amount of mechanical energy from the hand to the bat, creating a fast BSV [41,42].

**Figure 6:**
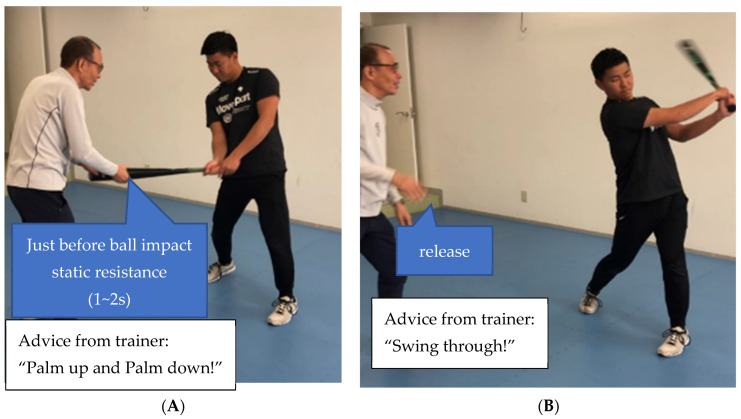
(**A**) Bat swing training (starting position). (**B**) Bat swing training (finish position).

I. Bat swing training (Figure 6A,B) is baseball-specific training that teaches a batter the proper starting position (Figure 6A) and receives the feeling of how to prepare for follow-through (Figure 6B). A trainer puts his/her hands on the tip of the bat (Figure 6A). Then, the trainer applies static resistance to the tip of the bat for 1~2 s. After that, the trainer release their hands from the bat, and the batter swings the bat through (Figure 6B). The trainer may advise the mechanical energy flow of a batter by checking static resistance before a ball impact and timing the release/extension of the bat.

**Training**—baseball-specific training, bat swing training (Figure 6A,B).

**Figure 7:**
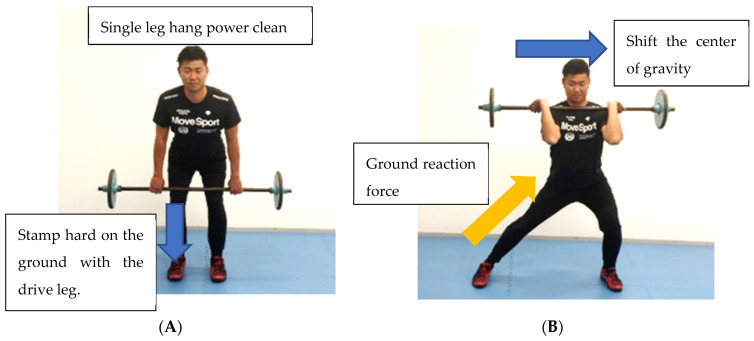
(**A**) Ground reaction force training (starting position). (**B**) Ground reaction force training (finish position).

II. Hang power clean exercise improves vertical power and enhances full body power [43]. When making this hang power clean exercise in baseball-specific batting motion training, it is necessary to transfer the momentum of the body obtained using the ground reaction force to the bat. The ground reaction force is obtained by strong lower limb stepping/stamping [43,44]. The purpose of ground reaction force training is two-fold; the first step is to stamp hard on the ground with the drive leg to obtain a large ground reaction force (Figure 7A). The second purpose is to transfer the ground reaction force to the stride leg by shifting the center of gravity sideways (Figure 7B). The force transmitted to the stride leg leads to fast pelvic rotation. This ground reaction force from the lower limbs creates a chain reaction to the trunk and upper limbs, leading from Slow and Middle BSV players to explosive Fast BSV players.

**Training**—Ground reaction force training (Figure 7A,B)

*Fast BSV groups:*

Fast BSV baseball players who have greater lean body mass have the highest BSV in the group (Table 4). As the level of competition increases, lean body mass increases, and the body becomes larger [18]. Increasing full-body lean mass is important to achieve even higher levels of competition.

I. The Big Three training (Figure 5) increases full-body lean mass and muscle strength. In addition, baseball-specific training aims to improve more explosive power, which is required for a successful bat swing.

**Figure 8:**
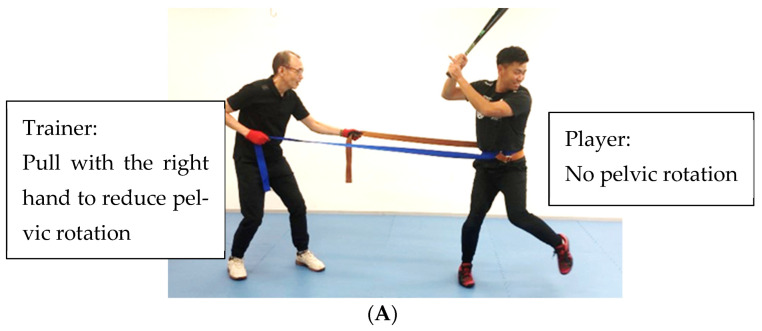

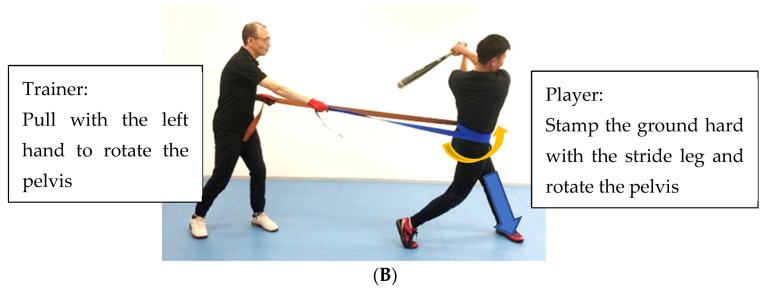
(**A**) Pelvic rotation training (starting position). (**B**) Pelvic rotation training (finish position).

II. Pelvic rotation training connects the ground reaction force obtained by the strong stamping stride leg to pelvic rotation (Figure 8A,B). In the starting position, the trainer pulls the rope to prevent the player’s pelvis from turning (Figure 8A). The trainer then pulls the rope with the opposite hand at the moment the player stamps the ground with their stride leg, causing the pelvis to rotate faster (Figure 8B). At this point, the trainer should remind the player to stamp hard with the stride leg and rotate the pelvis.

III. Scapular abduction/adduction switch training

**Figure 9:**
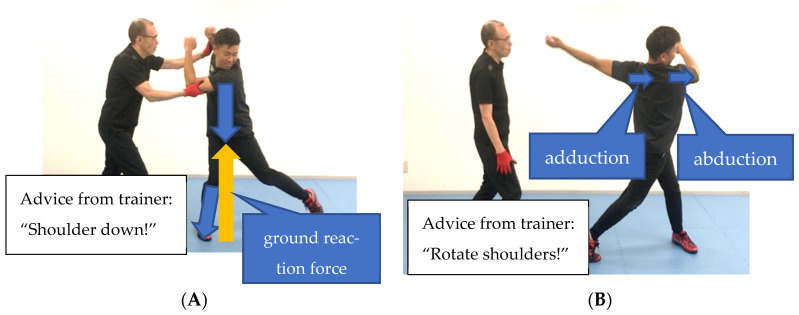
(**A**) Scapular abduction/adduction switch training (starting position). (**B**) Scapular abduction/adduction switch training (finish position).

The torso is connected to the upper limbs with the scapula. Scapular abduction/adduction switch training transfers energy from the lower limbs and pelvic rotation to the upper limbs (Figure 9A,B). In the starting position, the shoulder on the bottom-hand side suppresses the ground reaction force returned by stamping with the driving leg (Figure 8A). The scapula, at this point, should be in abduction on the bottom-hand side and adduction on the top-hand side. In the finishing position, the scapula is quickly replaced, and the body rotates (Figure 8B). The scapula, at this point, should be in adduction on the bottom-hand side with abduction on the top-hand side.

### Strengths and Limitations

The strength of this study is that it examines the correlation between detailed physical and fitness data and BSV. Based on these results, we propose baseball-specific training that is designed to improve BSV for collegiate baseball players. We believe the proposed baseball-specific training could be more motivational to baseball players than other basic training. Despite our pioneering applications to baseball players, our study has several limitations. First, the biggest limitation of this study refers to the power of statistical analysis. Although our correlations were statistically significant, these results were not powered to detect causation between anthropometric, physical, and BSV. Second, the sample size of our study can be considered a limitation because it is not representative of the study population. For example, in our study, there were a few players with weak grip strength but fast BSV who had a high-hitting technique different from the Slow and Middle groups. Third, the physical performance assessment protocols had some limitations, particularly regarding the number of trials that we used in this study. For example, we set one trial for a 30 m sprint testing and two trials for backward overhead medicine ball throwing based on our experiences and prior research in the literature. However, this number of trials may not reach each participant’s stable testing score [. This affects our physical performance assessment data. In addition, although the participants were able to perform all testing during the same study period, some of the participants performed a greater volume, intensity, and frequency of resistance training compared to other players. We had no control over their training prior to our study. Finally, BSV is often associated with a greater overall total hitter’s performance (i.e., batting average) [5]. However, this parameter was not assessed in this study. Therefore, it remains unknown how muscular strength improvement affects players with greater hitting proficiency. Thus, it is valuable to follow collegiate baseball players longitudinally regarding the BSV and anthropometric and physiological variables. In the future, more detailed factors for improving BSV are needed. For this reason, we would like to further increase the number of participants and conduct multivariate analyses to examine the factors necessary to improve BSV and confirm which of our results were not sufficiently represented in this study.

## 5. Conclusions

This study investigated the relationship between anthropometric and physiological variables alongside BSV in collegiate baseball players. Weak significant relationships were found between anthropometry and physiological performance variables and BSV. Multiple regression analysis showed the association of full-body lean body mass and back muscle strength. In addition, a multiple comparison test was performed with BSV divided into three parts (i.e., Fast, Middle, and Slow). The Fast BSV group had significantly greater full-body lean body mass, grip strength, back muscle strength, and backward overhead medicine ball throwing compared to the Slow BSV group. Based on these findings, we propose the combination of weight training and hitting as part of baseball-specific training protocols for improving BSV.

## Figures and Tables

**Table 1:** Anthropometric, batting performance, and physical performance tests (n = 78, mean ± SD).

Variables		Mean ± SD
**Batting Performance**		
BSV (km/h)		105.2 ± 6.1
**Anthropometric**		
Height (cm)		173.5 ± 5.0
Body mass (kg)		73.0 ± 7.7
Body mass index (kg/m^2^)		24.2 ± 2.2
Lean body mass (kg)		61.4 ± 4.8
Upper limb lean body mass (kg)	Top hand (dominant)	3.3 ± 0.3
	Bottom hand (non-dominant)	3.3 ± 0.3
Lower limb lean body mass (kg)	Drive leg	10.2 ± 0.7
	Stride leg	10.2 ± 0.7
Torso lean body mass (kg)		31.0 ± 2.6
**Physical performance**		
Hand grip (kg)	Top hand (dominant)	47.1 ± 5.1
	Bottom hand (non-dominant)	48.0 ± 5.5
Back muscle strength (kg)		144.7 ± 20.9
Standing long jump (cm)		228.2 ± 30.9
30 m sprint (s)		4.4 ± 0.2
Backward overhead medicine ball throwing (m)		13.0 ± 1.5

**Table 2:** Correlation between BSV and each measurement.

Variables	r	*p*-Value
**Anthropometric**		
Height (cm)	0.344	0.002
Body mass (kg)	0.500	<0.001
Body mass index (kg/m^2^) *	0.338	0.002
Lean body mass (kg)	0.542	<0.001
Upper limb lean body mass (kg)		
Top hand (dominant)	0.594	<0.001
Bottom hand (non-dominant)	0.495	<0.001
Lower limb lean body mass (kg)		
Drive leg	0.512	<0.001
Stride leg	0.491	<0.001
Torso lean body mass (kg)	0.526	<0.001
**Physical performance**		
Hand grip(kg)		
Top hand(dominant)	0.335	0.003
Bottom hand(non-dominant)	0.329	0.003
Back muscle strength (kg)	0.396	<0.001
Standing long jump (cm) *	0.170	0.138
30 m sprint (s)	−0.079	0.493
Backward overhead medicine ball throwing (m) *	0.289	0.010

* Spearman’s rank correlation coefficient.

**Table 3:** Multiple regression analyses for factors influencing BSV.

Variable	Unstandardized Coefficients		Standardized Coefficients	*p*-Value	VIF
	B	SE	β		
(Constant)	54.165	8.224		<0.001	
Lean body mass	0.549	0.140	0.430	<0.001	1.371
Hand grip					
Top hand	0.262	0.157	0.219	0.098	1.955
Bottom hand	−0.086	0.157	−0.077	0.583	2.250
Back muscle strength	0.067	0.031	0.230	0.032	1.268
Backward overhead medicine	−0.050	0.447	−0.012	0.912	1.344
Ball Throwing					

**Table 4:** Anthropometric and physical performance comparisons among 3 levels of BSV.

Variables	Fast (n = 26)	Middle (n = 26)	Slow (n = 26)
**Batting Performance**			
BSV	112.0 ± 4.0 km/h	104.9 ± 1.4 km/h	98.8 ± 2.8 km/h
**Anthropometric**			
Age	19.4 ± 1.0 years	19.5 ± 1.0 year	19.3 ± 0.9 year
Height	176.0 ± 4.4 cm ¶	172.9 ± 5.3 cm	171.7 ± 4.3 cm
Body mass	77.7 ± 7.3 kg †¶	72.2 ± 6.3 kg	69.1 ± 6.8 kg
Body mass index	25.1 ± 2.4 kg/m^2^ §	24.1 ± 1.6 kg/m^2^	23.4 ± 2.2 kg/m^2^
Lean body mass	64.6 ± 4.1 kg †¶	61.1 ± 4.1 kg	58.6 ± 4.2 kg
Upper limb lean body mass			
Top hand(dominant)	3.5 ± 0.3 kg ¶	3.3 ± 0.2 kg §	3.1 ± 0.3 kg
Bottom hand(non-dominant)	3.4 ± 0.2 kg †¶	3.3 ± 0.3 kg	3.1 ± 0.2 kg
Lower limb lean body mass			
Drive leg	10.6 ± 0.6 kg †¶	10.3 ± 0.7 kg	9.8 ± 0.7 kg
Stride leg	10.6 ± 0.7 kg ¶	10.3 ± 0.7 kg	9.8 ± 0.7 kg
Torso lean body mass	32.8 ± 2.4 kg †¶	30.6 ± 2.2 kg	29.6 ± 2.2 kg
**Physical performance**			
Hand grip			
Top hand(dominant)	49.2 ± 4.8 kg ¶	48.7 ± 4.6 kg ¶	43.6 ± 3.7 kg
Bottom hand(non-dominant)	50.5 ± 4.8 kg ¶	49.0 ± 5.4 kg §	44.6 ± 4.4 kg
Back muscle strength	155.2 ± 17.5 kg ¶	143.7 ± 17.7 kg	135.0 ± 21.9 kg
Standing long jump	235.0 ± 16.8 cm	233.6 ± 16.0 cm	216.1 ± 45.9 cm
30 m sprint	4.34 ± 0.19 s	4.33 ± 0.19 s	4.43 ± 0.16 s
Backward overhead medicine ball throwing	13.7 ± 1.7 m §	12.9 ± 1.4 m	12.3 ± 1.0 m

† *p* < 0.05 compared with Middle group, § *p* < 0.05 compared with Slow group, ¶ *p* < 0.01 compared with Slow group.

## Data Availability

The principal investigator (kay.hongu@gmail.com) have full access to the study data and takes responsibility for the integrity of the data and accuracy of the data analysis. The data will be available upon reasonable request to the principal investigator.
